# Whole-Genome *Yersinia* sp. Assemblies from 10 Diverse Strains

**DOI:** 10.1128/genomeA.01055-14

**Published:** 2014-10-23

**Authors:** H. E. Daligault, K. W. Davenport, T. D. Minogue, K. A. Bishop-Lilly, S. M. Broomall, D. C. Bruce, P. S. Chain, S. R. Coyne, K. G. Frey, H. S. Gibbons, J. Jaissle, G. I. Koroleva, J. T. Ladner, C.-C. Lo, C. Munk, G. F. Palacios, C. L. Redden, C. N. Rosenzweig, M. B. Scholz, S. L. Johnson

**Affiliations:** a Los Alamos National Laboratory (LANL), Los Alamos, New Mexico, USA; b Diagnostic Systems Division (DSD), United States Army Medical Research Institute of Infectious Diseases (USAMRIID), Fort Detrick, Maryland, USA; c Naval Medical Research Center (NMRC)-Frederick, Fort Detrick, Maryland, USA; d Henry M. Jackson Foundation, Bethesda, Maryland, USA; e United States Army Edgewood Chemical Biological Center (ECBC), Aberdeen Proving Ground, Aberdeen, Maryland, USA; f Center for Genome Sciences (CGS), USAMRIID, Fort Detrick, Maryland, USA

## Abstract

*Yersinia* spp. are animal pathogens, some of which cause human disease. We sequenced 10 *Yersinia* isolates (from six species: *Yersinia enterocolitica*, *Y. fredericksenii*, *Y. kristensenii*, *Y. pestis*, *Y. pseudotuberculosis*, and *Y. ruckeri*) to high-quality draft or complete status. The genomes range in size from 3.77 to 4.94 Mbp.

## GENOME ANNOUNCEMENT

*Yersinia* is a genus of Gram-negative facultative anaerobes belonging to the *Enterobacteriaceae* family, best known for its three main pathogens, *Yersinia enterocolitica*, *Y. pestis*, and *Y. pseudotuberculosis*. The genus was first described in 1894 by Alexandre Yersin (1), who isolated *Y. pestis* during the third plague pandemic. Generally, *Yersinia* spp. cause animal infections and humans are only incidental hosts (2). *Y. enterocolitica* and *Y. pseudotuberculosis* are both enteric pathogens while *Y. pestis* generally results in lymphadenitis (bubonic plague) and is derived from *Y. pseudotuberculosis* (2, 3). In this study, we sequenced and assembled 10 *Yersinia* strains, including 7 isolates of these 3 pathogenic species and 3 additional congeners.

High-quality genomic DNA was extracted from purified isolates of each strain using a Qiagen Genomic-tip 500 at the USAMRIID Diagnostic Systems Division (DSD). Specifically, 100-mL bacterial cultures were grown to stationary phase and nucleic acid was extracted per the manufacturer’s recommendations with one minor variation. For BSL3 *Yersinia pestis*, all cultures were lysed overnight to ensure sterility of the resulting extracted material. If sterility was not achieved, the nucleic acid was passed through a 0.45-µM filter and rechecked for viable organisms before removal from the BSL3 suite.

Sequence data for each draft genome were generated using a combination of Illumina and 454 technologies (4, 5). For each genome, we constructed and sequenced an Illumina library of 100-bp reads at high coverage (ranging from 119 to 733 bp) and a separate 454 library of long-insert paired-end reads (insert sizes ranging from 7.10 to 10.3 kb with 8- to 57-fold genome coverage). The two data sets were assembled together in Newbler (Roche) and the consensus sequences computationally shredded into 2-kbp overlapping fake reads (shreds). The raw reads were also assembled in Velvet and those consensus sequences computationally shredded into 1.5-kbp overlapping shreds (6). Draft data from all platforms were then assembled together with Allpaths, and the consensus sequences were computationally shredded into 10-kbp overlapping shreds (7). We then integrated the Newbler consensus shreds, Velvet consensus shreds, Allpaths consensus shreds, and a subset of the long-insert read pairs using parallel Phrap (High Performance Software, LLC). Possible misassemblies were corrected and some gap closure accomplished with manual editing in Consed (8–10).

Automatic annotation for each genome utilized an Ergatis-based workflow at LANL with minor manual curation. Each genome is available in NCBI (accession numbers are listed in Table 1) and raw data can be provided upon request. In-depth comparative analyses of these and other genomes are under way and will be published in subsequent reports.

**TABLE 1:** Strain-identifying information and basic statistics on assemblies and annotations

Species and strain	Alternate strain name	Accession no. (structure)	Size (bp) (% G+C content)	No. of CDS^*b*^	No. of rRNA genes	No. of tRNA genes	Plasmid^*a*^		
							pMT (pFra)	pPCP (pPst)	Pgm
*Yersinia enterocolitica*									
ATCC 9610	NCTC_12982	JPDV00000000 (1 scaffold, 7 contigs)	4,537,953 (47.3)	4,084	22	81	−	−	−
DATR	YE1013	JPDU00000000 (2 scaffolds, 3 contigs)	4,645,698 (47.3)	4,217	19	79	−	−	+
E265	YE1012	JPDW00000000 (3 scaffolds, 57 contigs)	4,694,189 (46.9)	4,268	18	78	−	−	−
YEA	NA^*c*^	JPDX00000000 (2 scaffolds; 82 contigs)	4,525,312 (47.0)	4,077	14	73	−	−	−
*Yersinia fredericksenii*									
ATCC 33641	CDC1461 to CDC1481	JPPS00000000 (2 scaffolds; 10 contigs)	4,941,072 (47.0)	4,363	22	80	−	−	−
*Yersinia kristensenii*									
ATCC 33639	CDC1459 to CDC1481	CP008955 (single closed chromosome)	4,442,328 (47.4)	3,946	22	82	−	−	+
*Yersinia pestis*									
CO92	YE0020CO92TA	JPMB00000000 (7 scaffolds; 59 contigs)	4,714,480 (47.6)	4,268	13	69	+	+	+
*Yersinia pseudotuberculosis*									
ATCC 4284	447	JPIY00000000 (4 scaffolds; 38 contigs)	4,768,560 (47.6)	4,190	15	78	−	−	+
ATCC 6904	NCTC 2476	CP008943 (single closed chromosome)	4,806,594 (47.6)	4,178	22	81	−	−	+
*Yersinia ruckeri*									
ATCC 29473	CDC 2396-61	JPPT00000000 (2 scaffolds; 15 contigs)	3,771,509 (47.4)	3,377	8	72	−	−	−

a −, not present; +, present.

b CDS, coding sequences.

c NA, not applicable.

### Nucleotide sequence accession numbers.

Genome accession numbers to public databases are listed in Table 1.
